# Going with the Grain of Cognition: Applying Insights from Psychology to Build Support for Childhood Vaccination

**DOI:** 10.3389/fpsyg.2016.01483

**Published:** 2016-09-30

**Authors:** Isabel Rossen, Mark J. Hurlstone, Carmen Lawrence

**Affiliations:** School of Psychology, University of Western AustraliaCrawley, WA, Australia

**Keywords:** backfire effect, information-deficit-model, intervention development, vaccination, vaccine hesitancy, vaccine confidence

## Abstract

Childhood vaccination is widely considered to be one of the most successful public health interventions. Yet, the effective delivery of vaccination depends upon public willingness to vaccinate. Recently, many countries have faced problems with vaccine hesitancy, where a growing number of parents perceive vaccination to be unsafe or unnecessary, leading some to delay or refuse vaccines for their children. Effective intervention strategies for countering this problem are currently sorely lacking, however. Here, we propose that this may be because existing strategies are grounded more in intuition than insights from psychology. Consequently, such strategies are sometimes at variance with basic psychological principles and assumptions. By going against the grain of cognition, such strategies potentially run the risk of undermining persuasive efforts to reduce vaccine hesitancy. We demonstrate this by drawing on key insights from cognitive and social psychology to show how various known features of human psychology can lead many intuitively appealing intervention strategies to backfire, yielding unintended and undesirable repercussions. We conclude with a summary of potential avenues of investigation that may be more effective in addressing vaccine hesitancy. Our key message is that intervention strategies must be crafted that go with the grain of cognition by incorporating key insights from the psychological sciences.

## Introduction

Childhood vaccination is a safe and effective way of reducing infectious diseases. Yet in many countries, there has been a decline in public confidence surrounding vaccination, leading some parents to delay or refuse vaccines for their children (Dubé et al., 2013). While rates of vaccination coverage remain generally high, such vaccine hesitancy is a significant cause for concern, since geographical clustering of vaccine refusal has recently contributed to outbreaks of diseases previously considered eradicated or controlled (Omer et al., 2008). In response to growing vaccine hesitancy, there has been an upsurge in research that has tracked the psychological, social, and contextual variables that contribute to vaccine hesitancy (Larson et al., 2014). However, while the drivers of vaccine hesitancy are well documented, effective intervention strategies for addressing the issue are sorely lacking (Sadaf et al., 2013; Dubé et al., 2015). Here, we argue that this may be because existing strategies have been guided more by intuition than by insights from psychology and by the erroneous assumption that humans act rationally (Kahneman and Tversky, 1979; Kahneman, 2003). We highlight the potential pitfalls of this approach by providing examples where interventions based on these assumptions have backfired; discuss why they backfired; and consider how such backfire effects might be averted. We propose that efforts to spur vaccination must go with the grain of cognition—they must be grounded in insights from psychology regarding how people think and act.

## Defining Vaccine Hesitancy

While there has been some disagreement in the literature regarding the exact definition of vaccine hesitancy, recently, in an effort to provide a standardized global definition, the World Health Organization SAGE working group on vaccine hesitancy settled on the following interpretation—“the delay or refusal of vaccination, despite the availability of vaccine services” (MacDonald, 2015). They identified three different drivers of vaccine hesitancy—*complacency* resulting from low risk perceptions of vaccine preventable diseases; a lack of *convenience* arising from insufficient access to vaccine services; and low *confidence* due to concerns about the safety of vaccines and the legitimacy of the services that deliver them (the “3Cs” model). While complacency and convenience are important reasons for vaccine hesitancy that merit psychological investigation, here we focus on hesitancy based on confidence (see Betsch et al., 2015, for a broader review), which encompasses uncertainty about the safety and effectiveness of vaccines, a lack of trust in the systems that deliver vaccines, and doubt about the motives of policy-makers who decide on the required vaccines. We focus on confidence because we regard it as a tipping point toward vaccine refusal or acceptance.

## Going *against* the grain of cognition

It is a shortcoming of many existing intervention strategies that they are grounded more in intuition than insights from psychology. Traditionally, for example, most strategies for raising confidence in immunization have been based on the so-called *Information Deficit Model* (IDM) of science communication. This model is predicated on the assumption that humans are rational and public misconceptions of science arise due to insufficient knowledge. On this approach, the solution to vaccine hesitancy is to provide hesitant parents with more scientific facts in order to plug the “knowledge gap” that is presumed to be the barrier preventing them from vaccinating their children. Given the widespread myths and misinformation surrounding childhood vaccination (Betsch et al., 2010; Kata, 2010; Jolley and Douglas, 2014), it is easy to see the intuitive appeal of this approach. The IDM is flawed, however—knowledge is rarely a good predictor of vaccination acceptance, and interventions that seek to inform or educate hesitant parents have little or no impact on vaccine confidence (Sadaf et al., 2013; Dubé et al., 2015; Jarrett et al., 2015).

A different approach is therefore required—one that is grounded in insights from psychology, rather than intuition. Intervention developers must recognize that there are complex cognitive, social, and affective processes that need to be taken into consideration when crafting interventions. As we show next, intervention strategies that disregard these complex psychological processes—such as those based on the IDM—may not merely be ineffective in their capacity to sway hesitant parents, they may even inadvertently increase their resistance to vaccination. Table 1 provides a concise overview of these backfire effects, which form the basis of our foregoing analysis. The fourth and fifth columns of the table summarize, respectively, the degree of evidence for theses backfire effects (+, weak evidence; ++, moderate evidence; +++, strong evidence) and whether this evidence is direct (viz. observed in a vaccination context), indirect (viz. observed in a context other than vaccination), or a combination of the two.

**Table 1:** **Summary of backfire effects**.

**Backfire effect**	**Brief description**	**References**	**Evidence?**	**Source**
Familiarity backfire effect	Repeated exposure to misinformation can increase an individuals familiarity with that misinformation, potentially leading them to assume it to be true.	Skurnik et al., 2005	+	Direct
Overkill backfire effect	When attempting to correct misinformation, conveying many counterarguments is cognitively taxing and can potentially lead people to reject the alternative explanation being advocated in favor of a simpler account based on the misinformation.	Schwarz et al., 2007	+	Indirect
Attitude polarization backfire effect	When confronted with belief-incongruent information, people tend to selectively call to mind evidence and arguments in opposition to this information, leading them to cling to their original beliefs even stronger than before.	Lord et al., 1979; Ditto and Lopez, 1992; Ditto et al., 2009; Kahan et al., 2010	+++	Direct + indirect
Sacred values backfire effect	When attitudes or beliefs are viewed as sacred—or as part of one's deeply held beliefs—monetary incentives or disincentives to change behavior tend to engender moral outrage and greater resistance to the behavior being advocated.	Tetlock, 2003; Ginges et al., 2007; Berns et al., 2012	++	Indirect
Social norms backfire effect	Highlighting an undesirable behavior as being regrettably frequent can backfire by communicating a descriptive norm signaling that the behavior is common, and therefore normal and approved of by others.	Cialdini et al., 1990; Cialdini, 2003; Cialdini et al., 2006	+++	Indirect
Group directed threat backfire effect	Messages that criticize a particular group—such as vaccine hesitant parents—can lead that group to show stronger group affiliation and greater resistance to out-group recommendations.	Ellemers et al., 2002	+++	Indirect
Fear appeals backfire effect	Persuasive messages that induce fear to encourage individuals to accept the messages' recommendations can potentially backfire by triggering defensive and avoidant responses.	Peters et al., 2013; Nyhan et al., 2014; Ruiter et al., 2014	++	Direct + indirect

### Cognitive constraints

Research in cognitive psychology shows that because of various biases of human memory, simply refuting vaccination myths and communicating scientific facts can backfire. For example, in order to debunk a myth, it seems logical to expose people to the myth so they know what you are referring to. Indeed, a common strategy for highlighting false information is to present myths juxtaposed with relevant facts. In one study examining the efficacy of such an approach, people were presented with a flyer displaying both myths and facts about the flu vaccine. Immediately after presentation, people could accurately separate the myths from the facts. Yet, 30 min later, most people had difficulties determining which of the statements about the flu vaccine were myths or facts (Skurnik et al., 2005). Dubbed the *familiarity backfire effect*, it seems that exposure to the myth can actually increase familiarity with the misinformation, paradoxically increasing the likelihood that people will recall it and assume it to be true (Lewandowsky et al., 2012).

Another example of how the mere mention of a vaccine myth can undermine informational interventions was reported by Nyhan et al. (2014). In their study, parents were presented with a passage taken from the Centers for Disease Control and Prevention correcting the widespread myth that the measles, mumps, and rubella (MMR) vaccine causes autism. Although myth-debunking reduced belief in the false claims, it also paradoxically decreased vaccination intent amongst those least favorable toward vaccination.

It is also possible to elicit the *overkill backfire effect* when attempting to correct misinformation. While it may seem intuitive to present many counterarguments to debunk a myth, processing many arguments is more cognitively taxing than processing a few, which renders it less likely that the information will be integrated into individuals' mental models, especially when compared to a simple and compelling myth (Schwarz et al., 2007; Cook and Lewandowsky, 2011; Lewandowsky et al., 2012).

### Social motivations

Attempts to change parental attitudes regarding vaccination by simply presenting people with scientific facts also overlook basic findings from social psychology. Psychologists have long demonstrated that people are motivated to defend and justify their pre-existing beliefs, even if those beliefs are in conflict with a wealth of evidence. Therefore, merely presenting evidence in favor of vaccination is unlikely to change attitudes because people engage in motivated reasoning—the tendency to search for information in support of—and disregard evidence in conflict with—one's prior beliefs (Lord et al., 1979; Ditto and Lopez, 1992; Ditto et al., 2009). Moreover, presenting information that clashes with people's worldviews can also lead to a backfire effect due to attitude polarization—when confronted with belief-incongruent information, people tend to selectively call to mind evidence and arguments in opposition to this information, leading them to cling to their original beliefs even stronger than before (Lord et al., 1979). There is some research demonstrating that people engage in motivated reasoning about vaccination. In a study of public acceptance of evidence regarding the HPV vaccine, people were more likely to discredit information about the safety and effectiveness of the vaccine when the information was framed in a way that clashed with their pre-existing worldviews (Kahan et al., 2010).

A similar line of research speaks to the caution that should be exercised when introducing monetary incentives or disincentives to advocate vaccination. Much public policy assumes that people are fundamentally creatures of the marketplace and can be encouraged to change their behavior if offered a financial motive. Yet, when behavior (such as refusing vaccination) is grounded in one's deeply held beliefs, it tends to be viewed as a moral rule that cannot be violated, rather than a preference that can be subject to cost-benefit analysis. Indeed, research shows that when people are asked to trade off their moral values for instrumental rewards they react with moral outrage and become even less likely to engage in the desired behavior (Tetlock, 2003; Ginges et al., 2007; Berns et al., 2012). These insights are particularly noteworthy in light of moves by various governments to withhold welfare payments or restrict access to other goods and services from parents who choose not to vaccinate their children. It may be that the introduction of monetary value to what is otherwise viewed as a moral issue has the potential to lead vaccine hesitant parents to become more entrenched in their beliefs. There is very recent evidence from an experimental setting showing that perceived coercion may have unintended consequences on vaccination rates. In a simulation of vaccine decision making, compulsory vaccination increased the level of moral outrage among those already opposed to vaccination and led vaccine hesitant individuals to be less likely to voluntarily vaccinate in subsequent iterations of the vaccine decision making paradigm (Betsch and Böhm, 2015).

Another pitfall of countless public health campaigns is the tendency to highlight a problem behavior as being regrettably frequent e.g., “parents are increasingly becoming distrustful of childhood vaccines.” This approach overlooks the basic tendency for people to act in accordance with social norms—people's perceptions of which behaviors are frequently performed. Therefore, highlighting the extent of a problem can backfire by inadvertently communicating a social norm drawing attention to the fact that the undesired behavior is engaged in by many people, and is therefore appropriate and normal (Cialdini et al., 1990; Cialdini, 2003; Cialdini et al., 2006). Indeed, studies in numerous contexts show that when people overestimate the prevalence and degree of social approval of undesirable behaviors (e.g., binge drinking) it increases the likelihood that they will engage in the undesirable social conduct (Schultz et al., 2007). It may be that recent public campaigns highlighting vaccine hesitancy as a growing problem have led people to overestimate the extent to which parents actually distrust vaccines, thereby decreasing their own confidence.

Insights from social identity theory further demonstrate the powerful influence of social context and group allegiances on behavior. People form an important part of their self-esteem from the social groups they belong to and identify with (Tajfel and Turner, 2004). When people feel that a group they identify with has been evaluated negatively, those who are highly committed to the group are likely to demonstrate even stronger group affiliation, display expressions of in-group loyalty, and a heightened willingness for collective action (Ellemers et al., 2002). This becomes problematic because it seems likely that social identities may be formed around opposition to vaccination, or at least more broadly around the adoption of an alternative lifestyle and questioning of the medical status quo (Attwell and Freeman, 2015; Leask, 2015). Therefore, interventions that explicitly deride vaccine hesitant parents or pit vaccinating parents against non-vaccinating parents may do more damage than good by threatening the group identities of those opposed to vaccination, thereby leading them to rally together and cling to their beliefs more strongly than before.

### Emotional responses

Another pervasive and intuitively appealing tactic for behavior change is the use of fear appeals. This approach is based on the assumption that if people fear the consequences of their risky behaviors, then they will be motivated to adopt safer alternatives. This logic seems particularly fitting in the context of vaccination, given that vaccines have become a “victim of their own success”—since the diseases prevented by vaccination have been reduced or eliminated, the dangers they pose are less salient than the risks of side-effects from vaccines.

The most recent and comprehensive meta-analytic assessment of the fear appeal literature concluded that fear appeals do work (Tannenbaum et al., 2015). However, fear appeals can potentially induce negative effects, since threatening health information can trigger defensive responses, such as risk denial, biased information processing, or inattention to health promotion messages (Peters et al., 2013). Moreover, such defensive and avoidant responses may occur disproportionately among those individuals most at risk from failing to engage in the desired health behavior (van't Riet et al., 2010). The possibility of a fear appeal backfiring may be increased if audiences perceive that they are unable to effectively adopt the messages' recommendations (Witte and Allen, 2000).

That fear appeals can sometimes be counterproductive is supported by the results of the study by Nyhan et al. (2014) who presented parents with fear appeals consisting of either a dramatic narrative of a child that contracts measles or a graphic picture of a child with measles. Paradoxically, both appeals led parents to express greater—rather than reduced—fear of side-effects from vaccination, compared with parents who were not exposed to a fear appeal. This result suggests it may be unwise to use threatening communications alone to combat vaccine hesitancy.

## Going *with* the grain of cognition

The number of possible backfire effects can paint a disheartening picture. Yet, there is also a wealth of psychological literature outlining optimal ways to design interventions to effectively shift behavior that may be relevant to vaccine hesitancy. Given what we know about how people's attitudes and decisions are systematically influenced by (1) how they remember information, (2) their group identities and deeply held beliefs, and (3) their emotional responses, effective interventions to build confidence in childhood vaccination can potentially be crafted to harness these fundamental aspects of human psychology.

For example, there are various debiasing techniques that can be used to correct myths surrounding vaccination (Cook and Lewandowsky, 2011). In order to avoid the familiarity backfire effect, it is best to begin by stating the facts; then introduce the myth; then debunk it; and finally replace the myth with a scientific fact. Crucially, the myth should never be repeated. Similarly, to avoid the overkill backfire effect, communicators should present a few—rather than many—counterarguments to a myth, since numerous counterarguments take more cognitive effort to process, thus reducing the potency of the correction. It is encouraging to note that these findings have been incorporated into recommendations by the European Centre for Disease Prevention and Control (ECDPC, 2014). However, there is currently no work that has directly tested these insights in the vaccination space, so their effectiveness in an experimental setting remains to be seen.

Other approaches suggest bypassing the facts altogether. For example, messages couched in terms of an individual's pre-existing beliefs and values are more likely to shift attitudes than those that are incongruent with their values (Kahan, 2010; Feinberg and Willer, 2013; Day et al., 2014). Opposition to vaccination tends to be based on a preference for a natural, alternative lifestyle, and the belief that governments should not intrude into one's personal life (Mills et al., 2005; Brown et al., 2010; Kata, 2010). It may be possible to redefine vaccination as congruent with such values. There is some promising work in this vein. A community based intervention found that framing vaccination as congruent with an alternative lifestyle led some parents lacking confidence in vaccination to feel more positively toward it (Attwell and Freeman, 2015). Future studies should further test the effectiveness of such an approach.

It may also be possible to take advantage of people's tendency to act in accordance with perceived social norms. In recent years, there has been an upsurge in social norms marketing campaigns which have harnessed the tendency for people to look to others for cues about the appropriate and correct ways to behave in order to promote socially desirable conduct. Interventions that seek to correct people's misperceptions of the prevalence and degree of social approval of deleterious behaviors such as drinking, smoking, energy consumption, littering, and gambling have proven very successful (Schultz et al., 2007; Moreira et al., 2009). Despite growing parental concern, vaccination rates are still high in most communities (90–95%). Communicating this high level of community endorsement may be an effective approach for leveraging support for vaccination. Research on social identity theory further suggests the conditions under which normative messages may be most potent. For example, if a counter-attitudinal message is communicated by an in-group source it is more likely to be accepted (Cohen, 2003).

Finally, it may be possible to use fear appeals productively—whilst at the same time avoiding the potential for such threatening communications to backfire—by incorporating a message component that induces positive emotions. Previous research suggests there are two ways in which this might be accomplished. Firstly, by incorporating a powerful self efficacy promoting element that persuades the message recipient that they are capable of adopting the fear appeal's recommendations (Ruiter et al., 2014; Tannenbaum et al., 2015), and secondly by getting message recipients to self-affirm by listing positive attributes about themselves or reflecting on their own cherished values (Sherman et al., 2000; Harris et al., 2007). It would be valuable to establish whether such augmentations of fear based messages might be effective motivators in the context of vaccine hesitancy.

## Concluding remarks

Too often, strategies to raise confidence in childhood vaccination ignore key insights from psychology. This is problematic because, as we have shown here, by disregarding such insights many common and intuitively appealing intervention strategies may inadvertently do more harm than good. Our strategies must go with the grain of cognition—they must be crafted in a way that acknowledges how people actually think and act, rather than how they ought to. In the same way that vaccination programmes rely on rigorous medical science, so too should vaccination interventions be informed by the best available evidence from the psychological sciences. Of course there are noteworthy instances where psychology has been incorporated into recommendations for communicating with vaccine hesitant parents (ECDPC, 2014; Betsch et al., 2015). Nevertheless, there remains a pressing need to build a strong empirical base of psychologically grounded intervention strategies that can inform efforts to counter vaccine hesitancy.

## Author contributions

All three authors contributed to the preparation of the paper. IR wrote the paper; MH contributed to the writing and editing of the paper; and CL commented on the different drafts.

### Conflict of interest statement

The authors declare that the research was conducted in the absence of any commercial or financial relationships that could be construed as a potential conflict of interest.
